# Cognition and Return to Work Status 2 Years After Breast Cancer Diagnosis

**DOI:** 10.1001/jamanetworkopen.2024.27576

**Published:** 2024-08-19

**Authors:** Marie Lange, Justine Lequesne, Agnes Dumas, Bénédicte Clin, Ines Vaz-Luis, Barbara Pistilli, Olivier Rigal, Christelle Lévy, Florence Lerebours, Anne-Laure Martin, Sibille Everhard, Gwenn Menvielle, Florence Joly

**Affiliations:** 1 ANTICIPE U1086 INSERM-UCN, Equipe Labellisée Ligue Contre le Cancer, Centre François Baclesse, Normandie Université UNICAEN, Caen, France; 2 Services Unit PLATON, Cancer and Cognition Platform, University of Caen Normandy, Caen, France; 3 Aix Marseille University, INSERM, IRD, ISSPAM, SESSTIM (Economic and Social Sciences of Health and Medical Information Processing), Marseille, France; 4 CHU de Caen, Service de santé au travail et pathologie professionnelle, F-14000 Caen, France; 5 INSERM, Gustave Roussy Institute, University Paris Saclay, Unit Molecular Predictors and New Targets in Oncology, Villejuif, France; 6 Medical Oncology Department, Gustave Roussy, Villejuif, France; 7 Care Support Department, Centre Henri Becquerel, Rouen, France; 8 Medical Oncology Department, Centre Henri Becquerel, Rouen, France; 9 Institut Normand du Sein, Centre François Baclesse, Caen, France; 10 Medical Oncology Department, Institut Curie, Saint Cloud, France; 11 UNICANCER, Paris, France; 12 Medical Oncology Department, CHU de Caen, Caen, France

## Abstract

**Importance:**

Return to work after breast cancer (BC) treatment depends on several factors, including treatment-related adverse effects. While cancer-related cognitive impairment is frequently reported by patients with BC, to date, no longitudinal studies have assessed its association with return to work.

**Objective:**

To examine whether cognition, assessed using objective and subjective scores, was associated with return to work 2 years after BC diagnosis.

**Design, Setting, and Participants:**

In a case series of the French Cancer Toxicities (CANTO) cohort, a study of patients with stage I to III BC investigated cognition from April 2014 to December 2018 (2 years’ follow-up). Participants included women aged 58 years or younger at BC diagnosis who were employed or looking for a job.

**Main Outcomes and Measures:**

The outcome was return to work assessed 2 years after BC diagnosis. Objective cognitive functioning (tests), cognitive symptoms, anxiety, depression, and fatigue were prospectively assessed at diagnosis (baseline), 1 year after treatment completion, and 2 years after diagnosis. Multivariable logistic regression models were used to explain return to work status at year 2 according to each cognitive measure separately, adjusted for age, occupational class, stage at diagnosis, and chemotherapy.

**Results:**

The final sample included 178 women with BC (median age: 48.7 [range, 28-58] years), including 37 (20.8%) who did not return to work at year 2. Patients who returned to work had a higher (ie, professional) occupational class and were less likely to have had a mastectomy (24.1% vs 54.1%; *P* < .001). Return to work at year 2 was associated with lower overall cognitive impairment (1-point unit of increased odds ratio [1-pt OR], 0.32; 95% CI, 0.13-0.79; *P* = .01), higher working memory (1-pt OR, 2.06; 95% CI, 1.23-3.59; *P* = .008), higher processing speed (1-pt OR, 1.97; 95% CI, 1.20-3.36; *P* = .01) and higher attention performance (1-pt OR, 1.63; 95% CI, 1.04-2.64; *P* = .04), higher perceived cognitive abilities (1-pt OR, 1.12; 95% CI, 1.03-1.21; *P* = .007), and lower depression (1-pt OR, 0.83; 95% CI, 0.74-0.93; *P* = .001) at year 2 assessment. Return to work at year 2 was associated with several measures assessed at baseline and year 1: higher processing speed (1-pt OR, 2.38; 95% CI, 1.37-4.31; *P* = .003 and 1.95; 95% CI, 1.14-3.50; *P* = .02), higher executive performance (1-pt OR, 2.61; 95% CI, 1.28-5.75; *P* = .01, and 2.88; 95% CI, 1.36-6.28; *P* = .006), and lower physical fatigue (10-pt OR, 0.81; 95% CI, 0.69-0.95; *P* = .009 and 0.84; 95% CI, 0.71-0.98; *P* = .02).

**Conclusions and Relevance:**

In this case series study of patients with BC, return to work 2 years after diagnosis was associated with higher cognitive speed performance before and after BC treatment. Cognitive difficulties should be assessed before return to work to propose suitable management.

## Key Points

**Question:**

Is cognition associated with return to work 2 years after breast cancer diagnosis?

**Findings:**

In this case series of 178 individuals with breast cancer in the French Cancer Toxicities trial cohort, return to work 2 years after breast cancer diagnosis was associated with higher cognitive speed performances before and after cancer treatment.

**Meaning:**

These findings suggest that cognitive difficulties among individuals treated for breast cancer should be assessed before return to work to propose suitable management.

## Teaser

This case series examines cognitive and performance status of women with breast cancer who have returned to work 2 years after diagnosis.

## Introduction

Due to improvement in the survival rate, employment after cancer treatment is an important issue for individuals with breast cancer (BC). Many women wish to return to work (RTW) after BC treatment in order to lead an active socioprofessional life, enjoy a better quality of life, and have financial security. Returning to work depends on several factors, such as working conditions, clinical characteristics (eg, cancer stage at diagnosis, comorbidities, and treatments), psychological symptoms (eg, anxiety and depression), and persistent cancer treatment-related adverse effects (eg, fatigue and pain).^1,2,3,4^

Cancer-related cognitive impairment (CRCI) is frequently reported by patients with BC: 50% or more report cognitive difficulties after adjuvant chemotherapy and 15% to 25% have objective cognitive decline.^5,6^ Cancer-related cognitive impairment mainly concerns processing speed, executive function, memory and attention difficulties.^7,8^ It can be assessed with self-reported measures (cognitive symptoms) or with cognitive tests (objective cognitive functioning). Systematic reviews have reported that cognitive symptoms are not associated with objective cognitive functioning but are frequently associated with anxiety, depression, and fatigue.^9,10^

Cancer-related cognitive impairment may be associated with difficulties with RTW. For example, 76% of patients with cognitive symptoms reported difficulties with RTW.^11^ Persons with cancer reported problems in planning and executing their work, reduced efficiency, and difficulties with memory, concentration, and word-finding.^12,13,14^

A systematic review reported that most studies have focused on the association between cognitive symptoms and work-related outcomes, such as work productivity and ability.^15^ Thus, higher levels of cognitive symptoms and fatigue 2 to 10 years after BC diagnosis were associated with lower future work ability.^16^ In persons with BC, cognitive symptoms 5 years after treatment were associated with poorer work ability, performance, and productivity.^17^

Few studies have assessed the association between objective cognitive functioning and work-related outcomes,^15^ and, to our knowledge, all of them were cross-sectional. Among them, a small case-control study observed that more individuals with cancer who had cognitive impairment (7 of 15) did not RTW 1 year after the first day of breast cancer–related sick leave in comparison with individuals without impairment (9 of 30), but the small sample size limited the results.^18^ In a larger sample of individuals with cancer who reported cognitive symptoms, small effect sizes were found for the association between work-related outcomes assessed 3 years after cancer diagnosis (work ability and physical functioning at work) and objective cognitive performances (mainly, overall cognition and motor performance).^19^

Among the studies on cognitive symptoms and work-related outcomes, to our knowledge, none assessed cognition before RTW. Furthermore, only 1 small study assessed the association between objective cognition and work status without cognitive assessment before RTW.^18^ Better understanding of the association between cognition and work status could allow vocational rehabilitation to be offered to individuals with CRCI after BC treatment, thereby preventing or minimizing the work-related difficulties associated with CRCI.

The main aim of this longitudinal case series study based on a large nationwide cohort was to examine whether cognition, assessed using objective and subjective scores, was associated with RTW 2 years after BC diagnosis in patients who were employed or looking for employment at diagnosis. Factors such as anxiety, depression, and fatigue were also considered. We hypothesized that cognitive functioning, assessed before RTW, would be associated with RTW.

## Methods

### Participants and Study Design

Data for this case series were obtained from a substudy of the nationwide French Cancer Toxicities (CANTO) cohort (n = 12 000) of women with stage I to III BC^20^, CANTO-Cog, which investigated cognitive functioning (from April 2014 to December 2018). CANTO-Cog recruitment details have been published.^21^ For the purpose of this study, we selected women who were working or looking for a job at diagnosis, aged 58 years or younger (to allow a 5-year delay before they reached the minimum retirement age) at the time of BC diagnosis and who had work-related characteristics at year 2. Furthermore, we selected only women who had undergone cognitive assessment at baseline and year 2 and for whom age data were available. The study was approved by the French regulatory authorities (Comité de protection des personnes Ile de France VII). All participants provided written informed consent and received financial compensation.

### Outcome and Measures

The outcome was RTW assessed 2 years after BC diagnosis. Return to work was dichotomized as RTW or no RTW (including sick leave, looking for a job, or disability status).

We investigated the following exposure variables. Cognitive functioning was assessed at diagnosis before treatment (baseline), approximately 1 year after diagnosis (3-6 months after treatment completion [year 1] and 2 years after diagnosis [year 2]). Results of neuropsychological tests and patient-reported outcomes (PROs) were analyzed according to a published method.^22^ Cognitive impairment was assessed for each cognitive domain according to International Cognition and Cancer Task Force recommendations^23^ (*z* score ≤−1.5 below healthy controls on 2 or more constitutive tests or *z* score ≤−2.0 below healthy controls on a single test) in order to create a score for overall objective cognitive impairment (defined by at least 2 impaired cognitive domains).^22,24^ Five cognitive domains were assessed: episodic memory, working memory, processing speed, attention, and executive function (eTable in Supplement 1).

Cognitive symptoms, anxiety, depression, and fatigue were also assessed at each visit by self-report validated questionnaires (PROs): Functional Assessment of Cancer Therapy–Cognitive-Function (FACT-Cog) (higher scores indicating lower cognitive symptoms), Hospital Anxiety and Depression Scale, and Fatigue-12 items.^25,26,27^

Using self-report questionnaires, work-related characteristics of patients with BC were collected at baseline (occupational class, parttime and full-time employment, and physical and psychosocial working conditions) and at year 2 (parttime and full-time employment, workplace accommodations, and work-life imbalance).^1^ Occupational class was based on the 6-category version of the French classification^28^: professionals and managers, technicians and associate professionals, clerks, self-employed individuals, manual workers, and farmers. Since numbers were limited, farmers were grouped with self-employed individuals. Occupational classes were also dichotomized as (1) professionals and managers, technicians, and associate professionals, and (2) clerks, self-employed individuals, manual workers, and farmers.

Sociodemographic data included age and educational level. Clinical data included Eastern Cooperative Oncology Group performance status, the Charlson comorbidity index, previous neurologic and psychiatric history, psychotropic medications, cancer stage at diagnosis, *ERBB2* (formerly *HER2*) status, and cancer treatments (surgery, chemotherapy, radiotherapy, hormonal therapy, and trastuzumab).

### Statistical Analysis

Baseline characteristics, cognitive measures, and PROs were described and compared according to RTW at year 2 by the *t* test for quantitative variables and the χ^2^ or Fisher exact test, if necessary, for qualitative variables. Two-sided hypothesis tests were constructed with a 5% level of significance. Multivariable logistic regression models were used to explain RTW at year 2 according to cognitive measures and PROs at year 2 (cross-sectional association), as well as PROs at baseline and year 1 (longitudinal association). One model per cognitive measure and/or PRO was constructed, each adjusted on age, occupational class (2 classes), cancer stage, and chemotherapy. Results of logistic regression models provided odds ratios (ORs) with 95% CIs and *P* values, with ORs greater than 1 indicating a positive association with RTW. For quantitative variables, ORs are presented with their corresponding unit of increase (eg, 1-pt OR for an increase of 1 point). Missing PRO data at follow-up were imputed using the scores obtained at the previous assessment.^22^ As there were few other missing data (<10%) (Table 1),^29^ only the missing PRO data were imputed. Statistical significance was set at *P* < .05. Statistical analyses were conducted with R software, version 4.2.1 (R Foundation for Statistical Computing).

**Table 1.** Baseline Demographic and Clinical Characteristics and Year 2 Work-Life Imbalance, Work Characteristics, and Overall Objective Cognitive Impairment

Characteristic	Participants, No. (%)		
	RTW (n = 141)	No RTW (n = 37)	*P* value
Socioeconomic data at baseline			
Age, median (range), y	48.5 (28-58)	50.4 (36-58)	.32
Education, median (range), y	14 (9-20)	12 (9-18)	.001
Educational level			
Low/middle	49 (34.8)	24 (64.9)	.002
High	92 (65.2)	13 (35.1)	
Occupational class			
Professionals and managers	49 (35.5)	6 (16.2)	.01
Technicians and associate professionals	34 (24.6)	6 (16.2)	
Clerks	43 (31.2)	15 (40.5)	
Self-employed (farmers, craftsmen, shopkeepers)	5 (3.6)	4 (10.8)	
Manual workers	5 (3.6)	4 (10.8)	
Unknown	2 (1.4)	2 (5.4)	
Missing	3 (2.1)	0	
Occupational class			
Professionals and managers, technicians, and associate professionals	83 (60.1)	12 (32.4)	.005
Other^a^	55 (39.9)	25 (67.8)	
Missing	3 (2.1)	0	
Working conditions at baseline			
Working hours			
Full-time employment	101 (74.8)	25 (78.1)	.87
Parttime employment	34 (25.2)	7 (21.9)	
Missing	6 (4.3)	5 (13.5)	
Work characteristics			
Strenuous	35 (26.1)	22 (68.8)	<.001
Missing	7 (4.9)	5 (13.5)	
Shift	11 (8.4)	9 (29.0)	.005
Missing	10 (7.1)	6 (16.2)	
Night, median (range)^b^	0 (0-8)	0 (0-30)	.13
Missing	26 (18)	12 (32)	
Weekly rest period of 48 consecutive hours	115 (85.2)	28 (84.8)	.90
Missing	6 (4.3)	4 (10.8)	
Independence on decision-making			
Totally agree	65 (48.9)	9 (28.1)	.002
Somewhat agree	52 (39.1)	13 (40.6)	
Tend to disagree or totally disagree	16 (12.1)	10 (31.3)	
Missing	8 (5.7)	5 (13.5)	
Work perceived as boring			
Totally agree	4 (3.0)	2 (6.5)	.06
Somewhat agree	6 (4.5)	4 (12.9)	
Tend to disagree or totally disagree	123 (92.5)	25 (80.6)	
Missing	8 (5.7)	6 (16.2)	
Clinical data			
Comorbidities, Charlson index: 0	111 (87.4)	28 (84.8)	>.99
1-2	15 (11.8)	4 (12.1)	
Missing	14 (9.9)	4 (10.8)	
ECOG 0	132 (98.5)	36 (100.0)	>.99
Missing	7 (5.0)	1 (2.7)	
Previous neurologic/psychiatric history	36 (25.5)	9 (24.3)	>.99
Psychotropic medications^c^	14 (9.9)	5 (13.5)	.74
Cancer stage≥II	87 (61.7)	30 (81.1)	.04
Missing	2 (1)	0	
*ERBB2*-positive	14 (10.0)	5 (13.5)	.75
Missing	1 (0.7)	0	
Treatments			
Breast-conserving surgery	114 (80.9)	17 (45.9)	<.001
Mastectomy	34 (24.1)	20 (54.1)	<.001
Chemotherapy	90 (63.8)	30 (81.1)	.07
Adjuvant radiotherapy	135 (95.7)	33 (89.2)	.25
Adjuvant hormonal therapy	120 (85.1)	33 (89.2)	.71
Trastuzumab	14 (9.9)	4 (10.8)	>.99
Work-life imbalance at year 2			
Equal importance to personal and professional life	45 (32.4)	5 (25.0)	.35
Personal life is more important	85 (61.2)	15 (75.0)	
Professional life is more important	9 (6.5)	0	
Missing	2 (1.4)	17 (45.9)	
Work characteristics at year 2			
Working hours			
Full-time employment	78 (56.5)	NA	NA
Parttime employment	60 (43.5)		
Missing	3 (2.1)		
Workplace accommodations			
Time employment	70 (51.1)	NA	NA
Missing	4 (2.8)		
Working schedules	41 (31.1)		
Missing	9 (6.4)		
Workload	31 (23.1)		
Missing	7 (5)		
Reasons for work activity			
Financial	102 (79.7)	NA	NA
Missing	13 (9.2)		
Well-being	111 (85.4)		
Missing	11 (7.8)		
Fear of seeing job disappear	19 (17.8		
Missing	34 (24.1)		

Abbreviations: ECOG, Eastern Cooperative Oncology Group; NA, not applicable; RTW, return to work.

^a^ Clerks, self-employed, manual workers, and farmers.

^b^ No. of time per months where work was performed at night for at least 2 hours, between 10 pm and 5 am.

^c^ Level 3 on the World Health Organization analgesic ladder, anxiolytics, antidepressant treatments, and hypnotics.

## Results

### Demographic and Clinical Characteristics

Among CANTO-Cog patients (n = 494; mean [SD] age, 53.7 [10.5] years; cancer stage≥II: 270 of 492 [54.9%]), 299 had a baseline cognitive assessment, were aged 58 years or younger, and were working or looking for a job at diagnosis (Figure). Of these, 51 had no year 2 cognitive assessment and 70 had unknown employment status at year 2. The final sample included 178 women with BC (median age, 48.7 [range, 28-58] years; there were no losses to follow-up), including 170 women who were working and 8 patients looking for a job at baseline. Of these, 141 were at RTW status at year 2. Among 37 women (20.8%) who did not RTW at year 2 (Table 1), 24 were on sick leave, 6 were looking for a job, 6 were disabled, and 1 had another status. Among the 8 patients looking for a job at baseline, only 1 was looking for a job at year 2. At baseline, 35 of all patients (19.6%) had overall cognitive impairment.

**Figure.**
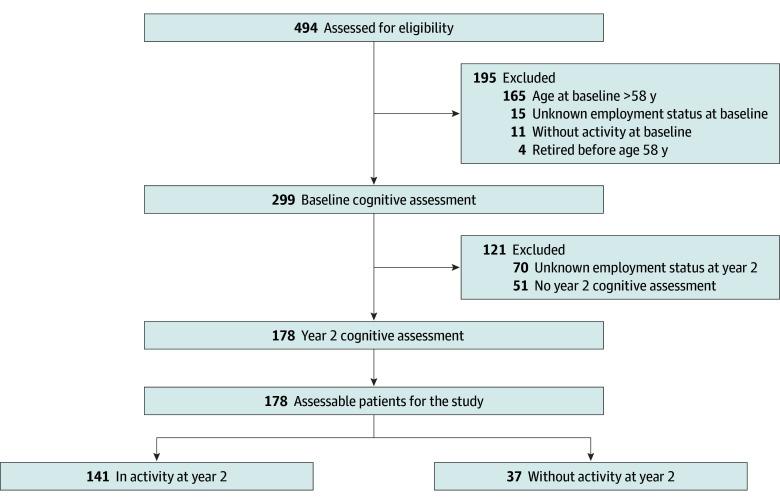
Patient Flowchart

### Baseline Characteristics of Patients According to RTW at Year 2 

At baseline, patients with RTW status at year 2 had a significantly higher occupational class (professionals, managers, technicians, and associated professionals, 60.1%) than those who did not RTW (32.4%) (*P* = .005) (Table 1). Their employment included less strenuous work (26.1% vs 68.8%; *P* < .001) and shift work (8.4% vs 29.0%; *P* = .005) and they were more independent in decision-making (48.9% vs 28.1%; *P* = .002).

Patients with RTW status were significantly less likely to have a mastectomy (24.1% vs 54.1%; *P* < .001) and had cancer greater than or equal to stage II less often (61.7% vs 81.1%; *P* = .04). There was no significant difference in other clinical data or treatment between groups.

Concerning cognition, patients with RTW status at year 2 had less baseline overall cognitive impairment than patients who did not RTW (16.3% vs 32.4%; *P* = .05) and higher baseline scores of episodic memory (0.24 vs −0.17; *P* = .05), processing speed (−0.08 vs −0.56; *P* = .001), executive function (0.14 vs −0.2; *P* = .001), and attention (−0.12 vs −0.55; *P* = .02) (Table 2). There was no significant difference between groups for episodic memory, working memory, and cognitive symptoms.

**Table 2.** Baseline Cognition and Patient-Reported Outcomes According to RTW Status

Assessment at baseline	Score, median (range)		
	RTW (n = 141)	No RTW (n = 37)	*P* value
Objective cognitive scores			
Overall cognitive impairment, No. (%)	23 (16.3)	12 (32.4)	.05
Episodic memory	0.24 (−3.2 to 1.2)	−0.17 (−2.7 to 1.2)	.05
Working memory, mean (SD)	−0.39 (0.67)	−0.63 (0.75)	.08
Processing speed	−0.08 (−1.9 to −1.2)	−0.56 (−2.6 to 0.9)	.001
Attention, mean (SD)	−0.12 (0.86)	−0.55 (0.99)	.02
Executive function	0.14 (−1.45 to 1.1)	−0.2 (−2.6 to 1.3)	.001
Patient-reported outcomes			
Cognitive symptoms: PCI	60 (14 to 72)	58 (26 to 72)	.89
Cognitive symptoms: PCA	20 (0 to 28)	19 (0 to 28)	.07
Cognitive symptoms: QOL	13 (0 to 16)	11 (1 to 16)	.25
Anxiety	8 (1 to 19)	9 (0 to 17)	.27
Depression	3 (0 to 16)	5 (0 to 16)	.07
Physical fatigue	20 (0 to 100)	33.3 (0 to 80)	.004
Emotional fatigue	11.1 (0 to 100)	33.3 (0 to 88.9)	.01
Cognitive fatigue	0 (0 to 100)	16.7 (0 to 83.3)	.12

Abbreviations: PCA, perceived cognitive abilities; PCI, perceived cognitive impairment; QOL, quality of life; RTW, return to work.

Patients with RTW status at year 2 reported less physical and emotional fatigue vs those who did not RTW (median scores: physical, 20 vs 33.3; *P* = .004; emotional, 11.1 vs 33.3; *P* = .01) (Table 2). There was no significant difference between groups for cognitive fatigue, anxiety, and depression.

### Year 2 Characteristics

At year 2, 14.2% (20 of 141) of patients with RTW status and 35.1% who did not RTW (13 of 37) had overall cognitive impairment (*P* = .007) (Table 3). There was no significant difference between groups in terms of work-life imbalance (Table 1). Among patients with RTW status at year 2, 43.5% (60 of 138) were parttime workers. Workplace accommodations mainly concerned reduced work time (51.1% [70 of 137]) and working schedules (31.1% [41 of 132]) (Table 1). The main reasons for RTW were well-being (85.4% [111 of 130]) and financial (79.7% [102 of 128]) (Table 1).

**Table 3.** Year 1 and Year 2 Cognition and Patient-Reported Outcomes According to RTW Status

Measure	Score, mean (SD)					
	Year 1^a^			Year 2		
	RTW at year 2 (n = 141)	No RTW at year 2 (n = 37)	*P* value	RTW at year 2 (n = 141)	No RTW at year 2 (n = 37)	*P* value
Objective cognitive scores						
Overall cognitive impairment, No. (%)	26 (19.0)	12 (35.3)	.07	20 (14.2)	13 (35.1)	.007
Episodic memory	−0.02 (0.75)	0.003 (0.79)	.88	0.10 (0.71)	−0.19 (1.02)	.11
Working memory	−0.17 (0.75)	−0.48 (0.76)	.04	−0.15 (0.75)	−0.54 (0.87)	.02
Processing speed	−0.19 (0.63)	−0.57 (0.98)	.04	−0.17 (0.74)	−0.58 (1.08)	.03
Attention	−0.03 (0.90)	−0.44 (0.92)	.02	0.02 (0.84)	−0.40 (0.92)	.01
Executive function	−0.01 (0.50)	−0.38 (0.62)	.002	0.07 (0.55)	−0.15 (0.55)	.03
Patient-reported outcomes						
Cognitive symptoms: PCI	52.7 (13.6)	50.8 (13.6)	.47	55.2 (12.9)	52.4 (12.0)	.21
Cognitive symptoms: PCA	18.1 (5.1)	16.4 (5.2)	.10	18.8 (5.0)	15.9 (5.04)	.004
Cognitive symptoms: QOL	12 (4.0)	10.6 (4.2)	.10	12.7 (3.82)	11.2 (4.26)	.07
Anxiety	6.77 (4.0)	7.28 (3.8)	.47	6.76 (3.71)	7.60 (3.86)	.24
Depression	3.63 (3.4)	4.44 (3.5)	.22	3.61 (3.28)	5.69 (4.13)	.007
Physical fatigue	32.3 (23.6)	41.3 (26.1)	.06	33.6 (23.9)	36.6 (25.4)	.52
Emotional fatigue	19.5 (24.1)	25.5 (25)	.19	21.0 (23.8)	22.5 (25.6)	.74
Cognitive fatigue	21.9 (24.4)	23 (23.7)	.80	17.6 (22.6)	23.4 (21.7)	.16

Abbreviations: PCA, perceived cognitive abilities; PCI, perceived cognitive impairment; QOL, quality of life; RTW, return to work.

^a^ Missing data at year 1, n = 7 of 178 (3.9%).

### Factors Associated With RTW at Year 2 

After adjustment for age, occupational class, cancer stage at diagnosis, and chemotherapy, cross-sectional analyses showed that RTW was associated with several measures assessed at year 2 (Table 4): lower overall cognitive impairment (1-pt OR, 0.32; 95% CI, 0.13-0.79; *P* = .01), higher working memory performance (1-pt OR, 2.06; 95% CI, 1.23-3.59; *P* = .008), higher processing speed performance (1-pt OR, 1.97; 95% CI, 1.20-3.36; *P* = .01), and higher attention performance (1-pt OR, 1.63; 95% CI, 1.04-2.64; *P* = .04), as well as higher perceived cognitive abilities (1-pt OR, 1.12; 95% CI, 1.03-1.21; *P* = .007; FACT-Cog) and lower depression (1-pt OR, 0.83; 95% CI, 0.74-0.93; *P* = .001).

**Table 4.** Cognition Scores and Patient-Reported Outcomes Associated With RTW Status at Year 2

RTW at year 2 measure^a^	1-pt OR					
	Longitudinal				Cross-sectional	
	Baseline score		Year 1 score^b^		Year 2 score	
	OR (95% CI)	*P* value	OR (95% CI)	*P* value	OR (95% CI)	*P* value
Objective cognition						
Overall impairment	0.40 (0.17-0.99)	.04	0.50 (0.21-1.24)	.13	0.32 (0.13-0.79)	.01
Episodic memory	1.40 (0.90-2.2)	.13	0.95 (0.55-1.58)	.84	1.43 (0.88-2.37)	.15
Working memory	1.54 (0.88-2.76)	.14	1.70 (0.98-3.07)	.06	2.06 (1.23-3.59)	.008
Processing speed	2.38 (1.37-4.31)	.003	1.95 (1.14-3.50)	.02	1.97 (1.20-3.36)	.01
Attention	1.55 (1.0-2.46)	.05	1.47 (0.95-2.30)	.09	1.63 (1.04-2.64)	.04
Executive function	2.61 (1.28-5.75)	.01	2.88 (1.36-6.28)	.006	1.67 (0.83-3.43)	.15
Cognitive symptoms						
PCI	1.00 (0.97-1.03)	.78	1.01 (0.98-1.03)	.68	1.02 (0.99-1.05)	.23
PCA	1.05 (0.98-1.12)	.19	1.06 (0.98-1.15)	.14	1.12 (1.03-1.21)	.007
QOL	1.06 (0.97-1.16)	.17	1.07 (0.98-1.18])	.14	1.09 (1.0-1.20)	.06
Anxiety/depression						
Anxiety	0.95 (0.87-1.03)	.21	0.95 (0.86-1.05)	.29	0.92 (0.83-1.02)	.12
Depression	0.92 (0.83-1.01)	.08	0.93 (0.83-1.04)	.18	0.83 (0.74-0.93)	.001
Fatigue						
Physical (10-pt OR)	0.81 (0.69-0.95)	.009	0.84 (0.71-0.98)	.02	0.93 (0.79-1.09)	.37
Emotional (10-pt OR)	0.87 (0.76-0.99)	.04	0.88 (0.75-1.03)	.09	0.95 (0.82-1.12)	.55
Cognitive (10-pt OR)	0.88 (0.75-1.04)	.12	0.98 (0.84-1.16)	.82	0.88 (0.74-1.04)	.12

Abbreviations: OR, odds ratio; PCA, perceived cognitive abilities; PCI, perceived cognitive impairment; QOL, quality of life; RTW, return to work.

^a^ Each measure corresponds to separate multivariable models adjusted for age, occupational class, cancer stage at diagnosis, and chemotherapy. For quantitative variables, ORs are presented with their corresponding unit of increase (eg, 1-pt OR indicates an increase of 1 point).

^b^ Missing data at year 1, n = 7 of 178 (3.9%).

Return to work at year 2 was associated with several measures assessed at baseline (Table 4): lower overall cognitive impairment (1-pt OR, 0.40; 95% CI, 0.17-0.99; *P* = .04), higher processing speed performance (1-pt OR, 2.38; 95% CI, 1.37-4.31; *P* = .003), higher executive function performance (1-pt OR, 2.61; 95% CI, 1.28-5.75; *P* = .01), lower physical fatigue (10-pt OR, 0.81; 95% CI, 0.69-0.95; *P* = .009), and lower emotional fatigue (10-pt OR, 0.87; 95% CI, 0.76-0.99; *P* = .04). Return to work at year 2 was associated with several measures assessed at year 1 (Table 4): higher processing speed performance (1-pt OR, 1.95; 95% CI, 1.14-3.50; *P* = .02), higher executive function performance (1-pt OR, 2.88; 95% CI, 1.36-6.28; *P* = .006), and lower physical fatigue (10-pt OR, 0.84; 95% CI, 0.71-0.98; *P* = .02).

Altogether, RTW status at year 2 was associated with processing speed performance at each time. Return to work at year 2 was associated with processing speed and executive performance and physical fatigue assessed at baseline and year 1. Return to work at year 2 was not associated with cognitive symptoms assessed at baseline or at year 1.

## Discussion

To our knowledge, this longitudinal case series is the first using a prospective cohort to examine whether cognition, including objective and subjective scores, is associated with RTW 2 years after BC diagnosis. Processing speed and executive function performance at diagnosis and after treatment completion were the main objective cognitive domains associated with RTW, as was physical fatigue. Two years after BC diagnosis, lower overall cognitive impairment; higher performance of working memory, processing speed, and attention; higher perceived cognitive abilities and lower depression were associated with RTW.

In this substudy of the CANTO study, we found that 20.8% of patients with BC who were working (n = 170) or looking for a job (n = 8) before cancer treatment had not returned to work 2 years after BC diagnosis. This is in line with the proportion found in the whole CANTO cohort^1^ and similar to that of a French national population-based survey.^30^

As found previously, RTW was associated with several factors, including socioeconomic (occupational class) and clinical (type of surgery) characteristics, working conditions (strenuous work, shift work, and low independence in decision-making), and psychological factors (depression).^31^ Furthermore, in line with previous reports, RTW at year 2 was associated with persistent cancer treatment-related adverse effects, such as physical fatigue, assessed before treatment and at year 1.^32^

To our knowledge, this case series is the first longitudinal study to observe that objective cognitive functioning is also associated with RTW status 2 years after BC diagnosis. Processing speed and executive function, assessed at diagnosis before cancer treatment and 1 year after cancer diagnosis, were associated with RTW at year 2. Thus, before treatment and after adjuvant treatment, patients with BC who had impaired cognitive speed and/or executive function, such as inhibition or flexibility, were those who had lower RTW status 2 years after diagnosis.

In addition, we found cross-sectional associations between work activity at year 2 and overall cognitive impairment, working memory, processing speed, and attention, perceived cognitive abilities, and depression. Cognitive symptoms assessed at baseline and at year 1 were not associated with RTW status at year 2. However, they were associated when they were assessed at the same time as RTW, such as at year 2. Previous studies have also observed that cognitive symptoms are higher among patients who do not RTW,^32^ but to our knowledge, no study has assessed cognitive symptoms before RTW.

Processing speed was associated with RTW status at year 2, regardless of assessment time. Studies have previously reported that this cognitive domain is frequently associated with RTW, particularly in patients with meningioma or after traumatic brain injury.^33,34^

We observed that more patients with objective cognitive impairment (35.1% [13 of 37]) at year 2 than without (14.2% [20 of 141]) did not RTW. This finding is consistent with a previous small study in which more individuals with cancer with (46.7% [7 of 15]) than without (30.0% [9 of 30]) cognitive impairment did not RTW.^18^ Nevertheless, this finding was not significant in that study, whereas the OR with adjustment was significant in ours.

It is important to better understand the association between cognition and work status, as cognitive difficulties are frequently associated with work difficulties in terms of productivity and ability.^15,16,17,35^ We found that objective cognitive domains, such as processing speed and executive function, assessed at diagnosis and the end of treatment were associated with RTW at year 2. As RTW after cancer is an important issue for individuals with BC, management of CRCI could be offered to patients whose processing speed and executive function have been impaired. Management for CRCI could be proposed by the occupational physician during sick leave or when RTW is planned. This timing issue requires further investigation, since a CRCI intervention before cancer treatment has been completed could compromise optimal management according to fatigue or other adverse effects induced by cancer treatment. Cognitive training has been shown to improve CRCI^36,37^ and experts recommend combining it with psychoeducation as part of a cognitive rehabilitation program.^38^ Furthermore, specific vocational rehabilitation, including training to increase processing speed and executive function, could be developed for women with cognitive difficulties who plan to RTW after BC treatments.

### Strengths and Limitations

A strength of this longitudinal case series based on a national prospective cohort of patients with BC is that it is, to our knowledge, the first to examine whether cognition assessed at diagnosis and the end of treatment is associated with RTW 2 years after diagnosis. It included a comprehensive assessment with both objective (based on International Cognition and Cancer Task Force recommendations)^23^ and subjective cognitive scores, psychological symptoms, and fatigue that could influence RTW.

The study has limitations. Several confounding factors were included in the analysis, but we cannot exclude residual confounding. In addition, we could not investigate trends in CRCI. Furthermore, patients with a high socioeconomic status may have been more likely to participate, as is often the case in studies based on self-reported data and in the CANTO cohort. In addition, this study did not have data on distances between home and work and did not include information about whether women wanted to RTW 2 years after their diagnosis (some may have reevaluated their priorities and chose not to RTW).

## Conclusions

The findings of this case series suggest that cognitive difficulties should be assessed before RTW to propose suitable management. Further work is required to understand the associations between cognition and work status in order to offer vocational rehabilitation to patients with cancer who have CRCI and to prevent work difficulties in productivity or ability associated with CRCI.

## Notes

Supplement 1. **eTable.** Neuropsychological Tests According to Cognitive Domains

Supplement 2.
Data Sharing Statement

## References

[1] Dumas A, Vaz Luis I, Bovagnet T, . Impact of breast cancer treatment on employment: results of a multicenter prospective cohort study (CANTO). J Clin Oncol. 2020;38(7):734-743. doi:10.1200/JCO.19.01726 31834818 PMC7048162

[2] Wang L, Hong BY, Kennedy SA, . Predictors of unemployment after breast cancer surgery: a systematic review and meta-analysis of observational studies. J Clin Oncol. 2018;36(18):1868-1879. doi:10.1200/JCO.2017.77.3663 29757686 PMC6804906

[3] Islam T, Dahlui M, Majid HA, Nahar AM, Mohd Taib NA, Su TT; MyBCC study group. Factors associated with return to work of breast cancer survivors: a systematic review. BMC Public Health. 2014;14(Suppl 3)(suppl 3):S8. doi:10.1186/1471-2458-14-S3-S8 25437351 PMC4251139

[4] Ruiz de Azua G, Kousignian I, Vaz-Luis I, . Sustainable return to work among breast cancer survivors. Cancer Med. 2023;12(18):19091-19101. doi:10.1002/cam4.6467 37602836 PMC10557874

[5] Lange M, Joly F, Vardy J, . Cancer-related cognitive impairment: an update on state of the art, detection, and management strategies in cancer survivors. Ann Oncol. 2019;30(12):1925-1940. doi:10.1093/annonc/mdz410 31617564 PMC8109411

[6] Ahles TA, Root JC, Ryan EL. Cancer- and cancer treatment-associated cognitive change: an update on the state of the science. J Clin Oncol. 2012;30(30):3675-3686. doi:10.1200/JCO.2012.43.0116 23008308 PMC3675678

[7] Joly F, Giffard B, Rigal O, . Impact of cancer and its treatments on cognitive function: advances in research from the Paris International Cognition and Cancer Task Force Symposium and update since 2012. J Pain Symptom Manage. 2015;50(6):830-841. doi:10.1016/j.jpainsymman.2015.06.019 26344551

[8] Fleming B, Edison P, Kenny L. Cognitive impairment after cancer treatment: mechanisms, clinical characterization, and management. BMJ. 2023;380:e071726. doi:10.1136/bmj-2022-071726 36921926

[9] Bray VJ, Dhillon HM, Vardy JL. Systematic review of self-reported cognitive function in cancer patients following chemotherapy treatment. J Cancer Surviv. 2018;12(4):537-559. doi:10.1007/s11764-018-0692-x 29728959

[10] Pullens MJ, De Vries J, Roukema JA. Subjective cognitive dysfunction in breast cancer patients: a systematic review. Psychooncology. 2010;19(11):1127-1138. doi:10.1002/pon.1673 20020424

[11] Lange M, Licaj I, Clarisse B, . Cognitive complaints in cancer survivors and expectations for support: results from a web-based survey. Cancer Med. 2019;8(5):2654-2663. doi:10.1002/cam4.2069 30884207 PMC6536919

[12] Von Ah D, Habermann B, Carpenter JS, Schneider BL. Impact of perceived cognitive impairment in breast cancer survivors. Eur J Oncol Nurs. 2013;17(2):236-241. doi:10.1016/j.ejon.2012.06.002 22901546

[13] Munir F, Burrows J, Yarker J, Kalawsky K, Bains M. Women’s perceptions of chemotherapy-induced cognitive side affects on work ability: a focus group study. J Clin Nurs. 2010;19(9-10):1362-1370. doi:10.1111/j.1365-2702.2009.03006.x 20500346

[14] Klaver KM, Duijts SFA, Engelhardt EG, . Cancer-related cognitive problems at work: experiences of survivors and professionals. J Cancer Surviv. 2020;14(2):168-178. doi:10.1007/s11764-019-00830-5 31768861 PMC7182611

[15] Tan CJ, Yip SYC, Chan RJ, Chew L, Chan A. Investigating how cancer-related symptoms influence work outcomes among cancer survivors: a systematic review. J Cancer Surviv. 2022;16(5):1065-1078. doi:10.1007/s11764-021-01097-5 34424498 PMC9489549

[16] Boelhouwer IG, Vermeer W, van Vuuren T. Work ability among employees 2-10 years beyond breast cancer diagnosis: late treatment effects and job resources—a longitudinal study. Work. 2023;74(3):1061-1076. doi:10.3233/WOR-211288 35527613

[17] Von Ah D, Storey S, Crouch A. Relationship between self-reported cognitive function and work-related outcomes in breast cancer survivors. J Cancer Surviv. 2018;12(2):246-255. doi:10.1007/s11764-017-0664-6 29222703

[18] Nieuwenhuijsen K, de Boer A, Spelten E, Sprangers MA, Verbeek JH. The role of neuropsychological functioning in cancer survivors’ return to work one year after diagnosis. Psychooncology. 2009;18(6):589-597. doi:10.1002/pon.1439 18942672

[19] Klaver KM, Duijts SFA, Geusgens CAV, . Neuropsychological test performance and self-reported cognitive functioning associated with work-related outcomes in occupationally active cancer survivors with cognitive complaints. J Cancer Surviv. 2024;18(2):412-424. doi:10.1007/s11764-022-01223-x 35776235

[20] Vaz-Luis I, Cottu P, Mesleard C, . UNICANCER: French prospective cohort study of treatment-related chronic toxicity in women with localised breast cancer (CANTO). ESMO Open. 2019;4(5):e000562. doi:10.1136/esmoopen-2019-000562 31555487 PMC6735667

[21] Lange M, Hardy-Léger I, Licaj I, . Cognitive impairment in patients with breast cancer before surgery: results from a CANTO cohort subgroup. Cancer Epidemiol Biomarkers Prev. 2020;29(9):1759-1766. doi:10.1158/1055-9965.EPI-20-0346 32611581

[22] Lange M, Lefevre Arbogast S, Hardy-Léger I, . Cognitive change in breast cancer patients up to 2 years after diagnosis. J Natl Cancer Inst. 2023;115(3):322-331. doi:10.1093/jnci/djac240 36571503 PMC9996221

[23] Wefel JS, Vardy J, Ahles T, Schagen SB. International Cognition and Cancer Task Force recommendations to harmonise studies of cognitive function in patients with cancer. Lancet Oncol. 2011;12(7):703-708. doi:10.1016/S1470-2045(10)70294-1 21354373

[24] Mandelblatt JS, Stern RA, Luta G, . Cognitive impairment in older patients with breast cancer before systemic therapy: is there an interaction between cancer and comorbidity? J Clin Oncol. 2014;32(18):1909-1918. doi:10.1200/JCO.2013.54.2050 24841981 PMC4050204

[25] Joly F, Lange M, Rigal O, . French version of the Functional Assessment of Cancer Therapy-Cognitive Function (FACT-Cog) version 3. Support Care Cancer. 2012;20(12):3297-3305. doi:10.1007/s00520-012-1439-2 22549504

[26] Zigmond AS, Snaith RP. The hospital anxiety and depression scale. Acta Psychiatr Scand. 1983;67(6):361-370. doi:10.1111/j.1600-0447.1983.tb09716.x 6880820

[27] Weis J, Tomaszewski KA, Hammerlid E, ; EORTC Quality of Life Group. International Psychometric Validation of an EORTC Quality of Life Module Measuring Cancer Related Fatigue (EORTC QLQ-FA12). J Natl Cancer Inst. 2017;109(5). Published online May 1, 2017. doi:10.1093/jnci/djw273 28376231

[28] Institut National del la Statistique et des Etudes ´ Economiques. Professions et categories socioprofessionnelles: PCS 2003. INSEE; 2019.

[29] Lee JH, Huber JC Jr. Evaluation of multiple imputation with large proportions of missing data: how much is too much? Iran J Public Health. 2021;50(7):1372-1380. 34568175 10.18502/ijph.v50i7.6626 PMC8426774

[30] La Vie Deux Ans Après Un Diagnostic de Cancer - De l’annonce à l’aprés Cancer, Études et Enquêtes. INCa (French Cancer Institute). 2014. Accessed July 11, 2024. https://www.e-cancer.fr/Expertises-et-publications/Catalogue-des-publications/La-vie-deux-ans-apres-un-diagnostic-de-cancer-De-l-annonce-a-l-apres-cancer

[31] Rai R, Malik M, Valiyaveettil D, Ahmed SF, Basalatullah M. Assessment of late treatment-related symptoms using patient-reported outcomes and various factors affecting return to work in survivors of breast cancer. Ecancermedicalscience. 2023;17:1533. doi:10.3332/ecancer.2023.1533 37138959 PMC10151075

[32] Schmidt ME, Scherer S, Wiskemann J, Steindorf K. Return to work after breast cancer: The role of treatment-related side effects and potential impact on quality of life. Eur J Cancer Care (Engl). 2019;28(4):e13051. doi:10.1111/ecc.13051 31033073

[33] Sekely A, Zakzanis KK, Mabbott D, . Long-term neurocognitive, psychological, and return to work outcomes in meningioma patients. Support Care Cancer. 2022;30(5):3893-3902. doi:10.1007/s00520-022-06838-5 35041087

[34] Sigurdardottir S, Andelic N, Wehling E, . Return to work after severe traumatic brain injury: a national study with a one-year follow-up of neurocognitive and behavioural outcomes. Neuropsychol Rehabil. 2020;30(2):281-297. doi:10.1080/09602011.2018.1462719 29667477

[35] Von Ah D, Storey S, Tallman E, Nielsen A, Johns SA, Pressler S. Cancer, cognitive impairment, and work-related outcomes: an integrative review. Oncol Nurs Forum. 2016;43(5):602-616. doi:10.1188/16.ONF.602-616 27541553

[36] Binarelli G, Duivon M, Joly F, Ahmed-Lecheheb D, Lange M. Cancer-related cognitive impairment: current perspectives on the management of cognitive changes following cancer treatment. Expert Rev Neurother. 2023;23(3):249-268. doi:10.1080/14737175.2023.2187288 36951414

[37] Chan RJ, McCarthy AL, Devenish J, Sullivan KA, Chan A. Systematic review of pharmacologic and non-pharmacologic interventions to manage cognitive alterations after chemotherapy for breast cancer. Eur J Cancer. 2015;51(4):437-450. doi:10.1016/j.ejca.2014.12.017 25623439

[38] Duivon M, Lange M, Binarelli G, . Improve the management of cancer-related cognitive impairment in clinical settings: a European Delphi study. J Cancer Surviv. Published online November 7, 2023. doi:10.1007/s11764-023-01436-8 37934312 PMC11502546

