# Supplementary material for: Cognition and Return to Work Status 2 Years After Breast Cancer Diagnosis
Source: JAMA Netw Open. 2024 Aug 19;7(8):e2427576. doi: 10.1001/jamanetworkopen.2024.27576 (PMC11333979; doi:10.1001/jamanetworkopen.2024.27576)
Supplement: Supplement 1. — eTable. Neuropsychological Tests According to Cognitive Domains [file jamanetwopen-e2427576-s001.pdf]

Supplementary Online Content

Lange M, Lequesne J, Dumas A, et al. Cognitive and return to work status 2 years after breast cancer diagnosis. *JAMA Netw Open*. 2024;7(8):e2427576.
doi:10.1001/jamanetworkopen.2024.27576

eTable. Neuropsychological Tests According to Cognitive Domains

This supplementary material has been provided by the authors to give readers additional information about their work.

© 2024 Lange M et al. *JAMA Network Open.*

eTable: Neuropsychological tests according to cognitive domains

Cognitive domains	Tests	Outcome measures
**Episodic memory**	HVLT	3 immediate free recall<br>Free delayed recall
**Working memory**	WAIS-III: Digit-span	Scaled score, forward<br>Scaled score, backward
	WAIS-III: Letter-number sequencing	Scaled score
	WMS-III: Spatial-span	Scaled score, forward<br>Scaled score, backward
**Processing speed**	TMT A	Time to complete and errors
	Stroop	Time to complete color and word cards
	WAIS-III: Symbol Search	Scaled score
**Attention**	d2 test	% of errors (F%)<br>Nb processed responses (GZ)<br>Nb of correct responses (KL)
**Executive function**	TMT B	Time to complete and number of perseverative
	Verbal fluency: Category (animal) and Letter P	errors<br>Total score over 2 min
	Stroop	Time to complete and number of non-corrected errors: interference card – color card

HVLT: Hopkins Verbal Learning test; WMS: Wechsler Memory Scale; TMT: Trail Making Test

© 2024 Lange M et al. *JAMA Network Open.*
