# Supplementary material for: Cognition and Return to Work Status 2 Years After Breast Cancer Diagnosis
Source: JAMA Netw Open. 2024 Aug 19;7(8):e2427576. doi: 10.1001/jamanetworkopen.2024.27576 (PMC11333979; doi:10.1001/jamanetworkopen.2024.27576)
Supplement: Supplement 2. — Data Sharing Statement [file jamanetwopen-e2427576-s002.pdf]

# Data Sharing Statement

Lange. Cognition and Return to Work Status 2 Years After Breast Cancer Diagnosis. *JAMA Netw Open*. Published August 19, 2024. doi:10.1001/jamanetworkopen.2024.27576

**Data**
**Data available:** Yes
**Data types:** Deidentified participant data
**How to access data:** c-gaudin@unicancer.fr
**When available:** With publication

**Supporting Documents**
**Document types:** None

**Additional Information**
**Who can access the data:** researchers whose proposed use of the data has been approved
**Types of analyses:** for a specified purpose
**Mechanisms of data availability:** after approval of a proposal and with a signed data access agreement
